# Brain Activations to Dyspnea in Patients With COPD

**DOI:** 10.3389/fphys.2020.00007

**Published:** 2020-01-24

**Authors:** Thomas Reijnders, Thierry Troosters, Wim Janssens, Rik Gosselink, Daniel Langer, Paul W. Davenport, Andreas von Leupoldt

**Affiliations:** ^1^Health Psychology, KU Leuven, Leuven, Belgium; ^2^Department of Rehabilitation Sciences, KU Leuven, Leuven, Belgium; ^3^Department of Chronic Disease, Metabolism and Aging, KU Leuven, Leuven, Belgium; ^4^Respiratory Division, University Hospital Leuven, Leuven, Belgium; ^5^Department of Physiological Sciences, University of Florida, Gainesville, FL, United States

**Keywords:** chronic obstructive pulmonary disease, respiratory related evoked potential, dyspnea, electro encephalogram, brain

## Abstract

We compared the perception and neural processing of respiratory sensations between 20 COPD patients and 20 healthy controls by means of respiratory-related evoked potentials (RREP) in the electroencephalogram (EEG). RREPs were induced by short inspiratory occlusions while 129-channel EEG was measured. COPD patients rated the occlusions as more intense and unpleasant (*p*’s < 0.001) and showed higher mean amplitudes for the RREP components P1 (*p* = 0.0004), N1 (*p* = 0.024), P2 (*p* = 0.019), and P3 (*p* = 0.018). Our results indicate that COPD patients demonstrate greater perception and neural processing of respiratory sensations, which presumably reflects the highly aversive and attention-demanding character of these sensations for COPD patients.

## Introduction

Chronic obstructive pulmonary disease (COPD) is a worldwide leading cause of morbidity and mortality, characterized by persistent and usually progressive airflow limitation (GOLD, 2017). The cardinal symptoms of COPD are respiratory sensations such as dyspnea (GOLD, 2017), which patients typically experience as highly unpleasant and frightening (Hutchinson et al., 2018). Consequently, many patients avoid these aversive sensations, often by reducing their physical activity levels, which contributes to further disease progression and reduced quality of life (Hutchinson et al., 2018). However, little is known about the neural mechanisms underlying the perception of respiratory sensations in COPD patients and whether these differ from healthy individuals. The few available studies using neuroimaging techniques with low temporal resolution such as fMRI have shown conflicting results. Yu et al. (2016) demonstrated higher brain activation during resistive-loaded breathing in COPD patients compared to healthy controls, whereas Esser et al. (2017) observed similar brain activation patterns between groups using comparable methodology. In contrast, studies in patients with other chronic symptoms (e.g., pain) that used neuroimaging techniques with high temporal resolution such as electroencephalography (EEG), clearly demonstrated greater neural processing of symptoms in patients compared to healthy controls (Lorenz and Garcia-Larrea, 2003). Therefore, by exploiting the high temporal resolution of respiratory-related evoked potentials (RREPs) in the EEG, the present study firstly investigated whether COPD patients would demonstrate not only greater perception, but also greater neural processing of respiratory sensations (higher amplitudes of the RREP components) compared to healthy individuals.

## Method

After providing written informed consent, twenty COPD patients and twenty age-matched control subjects without a history of respiratory disease were tested (ethical approval: B322201525306) (Table 1). Sample size was calculated based on results from previous studies in healthy subjects that used RREP’s to investigate the neural processing of respiratory sensations (e.g., 6). As previously described in more detail (Herzog et al., 2018), subjects respired through a breathing circuit with the inspiratory port connected to a pneumotachograph (Hans Rudolph, Shawnee, OK, United States) and occlusion device (Aspire, Gainesville, FL, United States). Respiratory variables and EEG data were continuously recorded (129-channel; sampling rate: 250 Hz; reference: Cz) (Philips EGI, Eugene, OR, United States). Subjects underwent two blocks of 3 min, during which inspiratory occlusions (complete stop of airflow for 600 ms) were randomly administered after the onset of inspiration every second to fourth breath via the occlusion device. After each block, the intensity and unpleasantness of occlusions were rated on a modified Borg scale ranging from 0 (“not noticeable/not unpleasant”) to 10 (“maximally imaginable intensity/unpleasantness”), followed by a 2 min-rest period.

**TABLE 1:** Overview.

**Variable**	**COPD patients**	**Control subjects**	***P* value**
	**(*n* = 20)**	**(*n* = 20)**	
Age (years)	63.4 (10.1)	67.7 (7.3)	ns
Sex (female/male)	(8/12)	(8/12)	ns
FEV1 (% predicted)	51 (23)	102 (13)	<0.001
FVC (% predicted)	88 (28)	105 (11)	0.013
FEV1/FVC (%)	48 (18)	78 (5)	<0.001
**Smoking status**			
Never smokers	1	7	
Ex-smokers	16	13	
Current smokers	3	0	
V_T_ (mL)	757 (247)	784 (239)	ns
V’ (L/s)	0.4 (0.1)	0.4 (0.1)	ns
P_Ima quiet breathing_ (cmH_2_O)	−1.1(0.3)	−1.0(0.3)	ns
P_Ima occlusions_ (cmH_2_O)	−5.7(2.9)	−3.3(1.3)	0.002
F (breaths/min)	16 (3)	15 (4)	ns
T_I_ (s)	1.9 (0.4)	2.2 (0.5)	0.032
Number of occlusions averaged	19 (4)	20 (5)	ns
Occlusion intensity (0–10)	3.5 (1.1)	2.0 (0.5)	<0.001
Occlusion unpleasantness (0–10)	3.7 (1.5)	2.1 (0.7)	<0.001
**RREP components mean amplitude (μV)**			
Nf	−1.65(1.86)	−1.07(1.07)	ns
P1	2.76 (1.69)	1.04 (1.07)	<0.001
N1	−4.15(3.01)	−2.32(1.69)	0.024
P2	2.61 (2.48)	0.86 (2.01)	0.019
P3	4.05 (3.02)	2.19 (1.32)	0.018
Anxiety (HADS-A)	7.6 (3.1)		
Health status (CRDQ)	71.6 (10.9)		
Symptom burden (mMRC)	2.2 (0.7)		

*Data are presented as mean (SD) unless otherwise indicated. FEV1, forced expiratory volume in 1 s; FVC, vital capacity; V_*T*_, tidal volume; V’, inspiratory flow; P_*Imax*_, inspiratory mouth pressure; F, breathing frequency; T_*I*_, inspiratory time; RREP, respiratory-related evoked potential; HADS-A, Hospital Anxiety and Depression Scale-Anxiety; CRDQ, Chronic Respiratory Disease Questionnaire; mMRC, Modified Medical Research Counsil Dyspnea Scale.*

The neural processing of respiratory sensations was measured by RREPs recorded from the EEG, which were elicited by the activation of mechanoreceptors due to the inspiratory occlusions (von Leupoldt et al., 2011). Early RREP components (<130 ms) Nf, P1 and N1 represent the first-order sensory processing of afferent respiratory signals to sensorimotor cortices. Later components (>150 ms) P2 and P3 reflect higher-order cognitive processing and are related to affective processing and motivated attention, with N1 being partly related to similar affective/attentional processes (Donzel-Raynaud et al., 2009; von Leupoldt et al., 2011; Herzog et al., 2018). EEG data were processed offline as previously described (von Leupoldt et al., 2011) using BESA Research 6.0 (BESA, Gräfelfing, Germany).

SPSS 24 (IBM, Armonk, NY, United States) was used for statistical analyses. Ratings of occlusion intensity and unpleasantness, respiratory variables and mean amplitudes for RREP components Nf, P1, N1, P2, and P3 were averaged across both experimental blocks and compared between groups (COPD vs. controls) using independent *t*-tests. Additionally, correlations (Pearson’s *r*) between mean amplitudes for RREP components, ratings and mouth pressure responses to occlusions were tested. The level of significance was *p* < 0.05.

## Results

**FIGURE 1:**
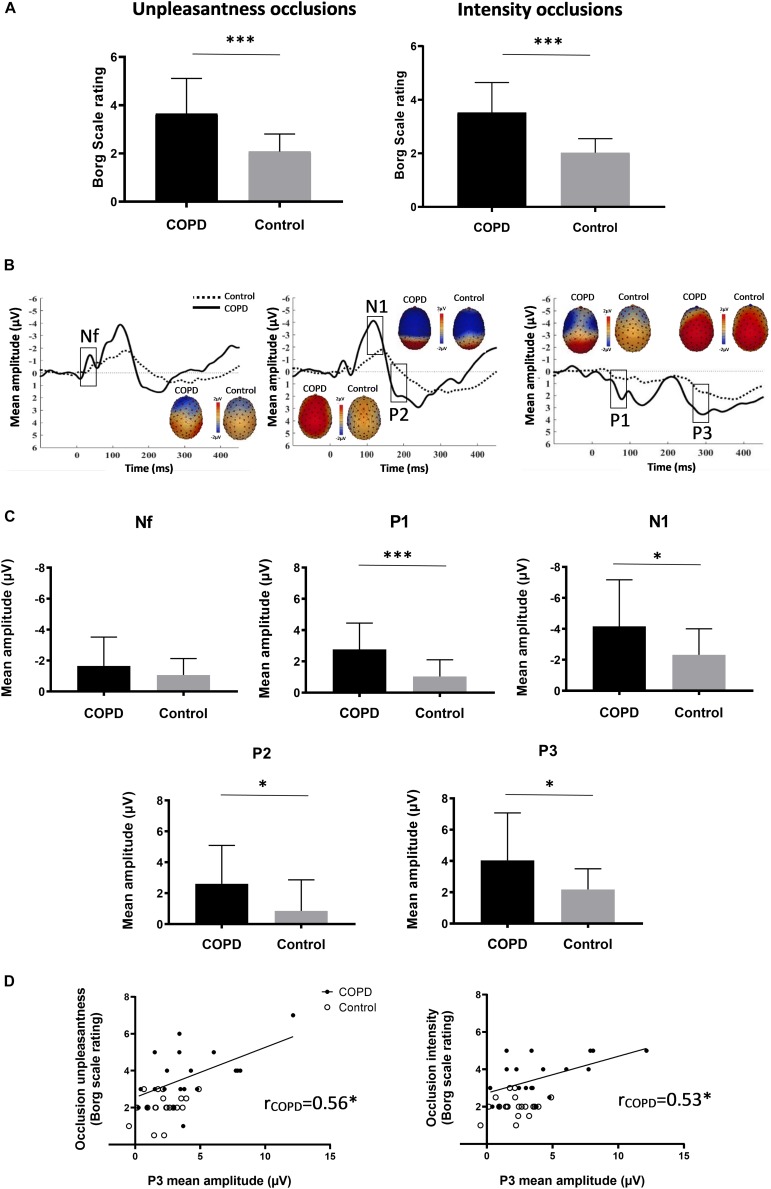
**(A)** Mean (±SD) ratings of occlusion intensity and unpleasantness for COPD patients and control subjects. **(B)** Averaged waveforms (μV) of the respiratory-related evoked potential for COPD patients and control subjects from sensors at frontal (Nf), centro-lateral (N1 & P2), and centro-parietal (P1 & P3) scalp positions. Based on previous reports (von Leupoldt et al., 2011), RREP components were identified as: Nf, negative peak – frontal region (latency: 25–50 ms); P1, positive peak – centro-parietal region (latency: 45–65 ms); N1, negative peak – centro-lateral region (latency: 85–125 ms); P2, positive peak – central region (latency: 160–230 ms); P3, positive peak – centro-parietal region (latency: 250–350 ms). RREP components and respective topographical areas are indicated on the figure. **(C)** Mean (±SD) amplitudes (μV) for RREP components Nf, P1, N1, P2, and P3 for COPD patients and control subjects. Mean amplitudes were calculated around each individual peak using a latency window of ±10 ms for Nf and P1, and ±20 ms for N1, P2, and P3. **(D)** Correlations between mean P3 amplitudes (μV) and Borg scale ratings of intensity and unpleasantness for COPD patients. ^∗^*p* < 0.05; ^∗∗∗^*p* < 0.001.

Chronic obstructive pulmonary disease patients rated the occlusions as significantly more intense and unpleasant compared to the control subjects (Figure 1A and Table 1). The mean number of averaged occlusions was comparable between groups (Table 1). Most respiratory variables did not differ between both groups (Table 1).

Most importantly, significant group differences in RREP components were observed with COPD patients showing greater mean amplitudes than control subjects for P1 (*p* = 0.0004), N1 (*p* = 0.024), P2 (*p* = 0.019), and P3 (*p* = 0.018). No significant difference was observed for Nf (*p* = 0.23) (Figures 1B,C; Table 1). Notably, higher ratings for occlusion intensity and unpleasantness were correlated with greater P3 amplitudes in COPD patients (*p*’s < 0.05), but not in healthy controls (Figure 1D). In COPD patients, mouth pressure responses to occlusions were larger (Table 1) and correlated with higher early P1 and N1 amplitudes (*r* = −0.69, *p* = 0.001; *r* = 0; 74, *p* = 0.0003), but not with later P2 and P3 (*r* = 0.17, *p* = 0.480; *r* = 0.03, *p* = 0.914) amplitudes, while no such relations were observed in controls.

## Discussion

The present results demonstrate that COPD patients show greater neural processing of respiratory sensations compared to matched control subjects without respiratory disease. This amplified neural processing in COPD patients, especially when related to later cognitive-affective processing stages, was correlated with greater perception of intensity and unpleasantness of these sensations, but not related to stimulus intensity quantified as mouth pressure responses. Given that earlier studies in healthy subjects have demonstrated that especially higher amplitudes of RREP components P2 and P3 reflect motivated attention for respiratory stimuli (von Leupoldt et al., 2011), the present findings most likely reflect the more aversive, frightening and motivationally relevant character of respiratory sensations for COPD patients, ultimately demanding greater neural processing capacities.

The present findings converge with previous EEG studies in patients suffering from other chronic aversive symptoms such as pain, which similarly demonstrated greater allocation of neural resources to their relevant symptoms than control subjects (Lorenz and Garcia-Larrea, 2003). Most importantly, the present results support some of the few previous neuroimaging findings in COPD patients using lower temporal resolution techniques. For example, using functional magnetic resonance imaging, Yu et al. (2016) have demonstrated that patients with COPD show higher activation in several brain areas including emotion-related brain areas compared to healthy controls during resistive load induced dyspnea.

Notably, previous studies demonstrated that harmless stimuli associated with aversive respiratory sensations (e.g., dyspnea-related verbal statements, visual cues signaling upcoming dyspnea) evoked increased neural processing in COPD patients, which was partly related to clinical outcomes in these patients (Herigstad et al., 2015, 2017; Esser et al., 2017). Together with the present findings, this supports the notion that increased attention to and neural processing of perceived as well as merely anticipated respiratory sensations might contribute to increased perception of dyspnea and potentiate subsequent avoidance of associated situations such as physical activities (von Leupoldt, 2017). This might contribute to progressive worsening of symptoms, reduced quality of life, and limited treatment efforts and might partly explain the often observed weak relationships between dyspnea ratings and level of lung function impairment (Müllerová et al., 2014). Subsequently, the present results suggest a potential neural target for pharmacological and/or non-pharmacological treatments, which clearly necessitates further research efforts.

This study has limitations related to the limited number of averaged occlusions per subject, warranting caution when interpreting lower amplitude components such as Nf and P1. Future studies should, therefore, increase the number of occlusions as well as compare subgroups of patients with different clinical characteristics. Such studies might benefit from controlling for additional measures of respiratory muscle mechanoreceptor activity including muscle strength, operating lung volumes and velocity of inspiratory muscle shortening, which could potentially influence early RREP components. Moreover, experimentally evoked short inspiratory occlusions represent only one quality of respiratory sensations restricting the generalizability of the findings to more long-lasting, real life sensations such as activity-induced breathlessness. Lastly, the data analysis was not performed blinded. However, we want to emphasize that the authors hold data integrity and ethical standards in high regard. In addition, several steps of the RREP analyses are semi-automatically performed, which limits potential biases by the analyzing person.

## Conclusion

In summary, this study demonstrated firstly with high temporal resolution EEG methodology that COPD patients show not only greater perception, but also greater neural processing of respiratory sensations, which presumably reflects the highly aversive and attention-demanding character of these sensations for COPD patients.

## Data Availability Statement

The datasets generated for this study are available on request to the corresponding author.

## Ethics Statement

The studies involving human participants were reviewed and approved by the Commissie Medische Ethiek, UZ KU Leuven (University Hospital KU Leuven) (B322201525306). The patients/participants provided their written informed consent to participate in this study.

## Author Contributions

TR, TT, WJ, RG, DL, PD, and AL contributed to the conception and design, the interpretation of data, the drafting the manuscript, and the manuscript revision. TR contributed to the data acquisition. TR and AL contributed to the data analysis, and had full access to all of the data in the study and took full responsibility for the integrity of the data and accuracy of the data analysis. All authors read and approved the final version.

## Conflict of Interest

The authors declare that the research was conducted in the absence of any commercial or financial relationships that could be construed as a potential conflict of interest.
